# Iron as an immunometabolic signal in infection and inflammation

**DOI:** 10.3389/fimmu.2026.1810718

**Published:** 2026-04-16

**Authors:** Alexander Hoffmann, Laura Homs Pérez, Günter Weiss

**Affiliations:** 1 Department of Internal Medicine II, Infectious Diseases, Immunology, Rheumatology, Medical University of Innsbruck, Innsbruck, Austria; 2 Christian Doppler Laboratory for Iron Metabolism and Anemia Research, Medical University of Innsbruck, Innsbruck, Austria

**Keywords:** host-pathogen interaction, immuno-metabolism, inflammation, innate and adaptive immunity, iron homeostasis, iron signaling, lysosomal iron, mitochondrial respiration

## Abstract

Iron availability is dynamically remodeled during infection and inflammation and has traditionally been interpreted within the framework of nutritional immunity, where iron sequestration restricts microbial growth. Increasing evidence, however, indicates that inflammatory iron redistribution has broader immunological consequences. Systemic and cell-intrinsic iron levels actively shape immune cell production, differentiation, metabolic configuration, and effector function across innate and adaptive immune compartments. Rather than uniformly suppressing immunity, inflammation-driven hypoferremia selectively biases hematopoiesis and immune output, while locally regulated cellular and subcellular iron handling determines microbe-specific immune effector functions. In addition, the availability of metabolically active iron further defines immunometabolic state by regulating mitochondrial respiration, tricarboxylic acid cycle activity, and redox balance, thereby shaping both immune cell function and the host-pathogen interface. Together, these observations support a view of iron as an instructive immunometabolic signal that integrates systemic regulation with cellular and organelle-specific programs during infection and inflammation. This review synthesizes recent evidence across these organizational scales, highlights emerging trade-offs between host defense and iron homeostasis, and discusses how mechanistic insight into iron-sensitive immune pathways may inform strategies to modulate inflammation and antimicrobial immune effector function.

## 1 Introduction

Iron is an essential micronutrient for both host and pathogen, supporting core processes such as energy metabolism, DNA synthesis, and redox homeostasis. At the cellular level, iron acts as a cofactor for multiple enzymes, including those involved in mitochondrial respiration, tricarboxylic acid (TCA) cycle activity, and DNA synthesis. It is also required for the formation of heme and iron-sulfur cluster-containing proteins (1). Because these processes are particularly critical for rapidly proliferating or metabolically active cells, a sufficient supply of iron for such cells and tissues is essential and multiple pathways are involved in systemic and cellular iron handling, trafficking and utilization. Comprehensive overviews of systemic and cellular iron metabolism and its regulation in health and disease have been excellently summarized elsewhere (2, 3). Key host and bacterial cellular iron handling systems discussed throughout this review are summarized in **Table 1**, providing an overview of the principal components addressed in subsequent sections. During infection and inflammation, host iron availability is dynamically remodeled, a response classically framed as nutritional immunity, in which iron sequestration limits microbial growth (4). While this concept captures the competitive aspect of host-pathogen interactions, it does not fully explain the broader immunological consequences of inflammatory iron redistribution.

**Table 1:** Major host and bacterial systems controlling iron acquisition, intracellular handling, and detoxification during infection.

Functional category	Mammalian cells	Bacteria
Iron uptake and acquisition	Transferrin receptor (TfR1) – receptor-mediated uptake of transferrin-bound ferric iron (Fe³^+^)	Siderophore systems – secretion of high-affinity chelators that scavenge Fe³^+^
	Divalent metal transporter 1 (DMT1) – transport of ferrous iron (Fe²^+^) across endosomal or plasma membranes	TonB-dependent receptors – energy-dependent uptake of Fe³^+^ complexes (e.g. siderophores or heme)
		FeoABC transporter – uptake of Fe²^+^
Intracellular iron handling	Ferritin – intracellular iron storage and buffering (Fe³^+^)	Ferritin-like proteins – intracellular iron storage (Fe³^+^)
	Nramp1 (SLC11A1) – export of Fe²^+^ from phagosomes to restrict pathogen access	
Iron export/detoxification	Ferroportin (FPN1) – cellular iron export (Fe²^+^)	Heme detoxification systems (e.g. HrtAB) – protection against heme toxicity

Mammalian cells regulate iron availability through transferrin-mediated uptake, intracellular storage in ferritin, and controlled export via ferroportin. Bacterial pathogens employ diverse strategies to acquire iron from the host environment, including siderophore secretion, TonB-dependent uptake of ferric iron complexes such as siderophores or heme, and ferrous iron transport via Feo systems. Additional mechanisms, such as heme detoxification systems, protect bacteria from heme-associated toxicity.

Although inflammatory iron sequestration is traditionally framed as a host defense strategy, emerging data indicate that iron handling is mechanistically linked to immune cell signaling and differentiation programs (3, 5). Variations in systemic and cellular iron levels influence immune cell development, activation thresholds, and effector capacity across both innate and adaptive compartments. These effects extend beyond metabolic limitation, indicating that iron-dependent regulatory programs are embedded within immune signaling and differentiation pathways. In this context, we use the term “immunometabolic signal” to describe situations in which changes in iron availability actively influence immune cell signaling, cellular metabolic pathways and their prioritization, and effector responses beyond iron’s classical role as a nutrient and enzymatic cofactor. Indeed, while iron is essential for numerous metabolic reactions, fluctuations in iron availability during infection and inflammation can themselves act as regulatory signals that shape immune cell behavior and the host-pathogen metabolic interface.

Importantly, inflammatory iron regulation is not uniform. Systemic iron sequestration coexists with locally controlled and cell-type-specific iron responses, resulting in divergent immune outcomes across tissues and immune compartments. Understanding iron as an immunometabolic signal therefore requires a perspective that integrates systemic regulation with cellular and intracellular iron handling.

Here, we discuss how iron availability acts as an immunometabolic signal that links iron homeostasis to immune regulation during infection and inflammation, with a primary focus on bacterial infections, where the impact of alterations in iron homeostasis on host-pathogen interactions has been extensively characterized. We highlight mechanisms by which iron shapes immune cell metabolism, differentiation, and effector function across organizational scales, and discuss emerging implications for host defense and inflammatory pathology.

## 2 Systemic iron redistribution as an immunoregulatory signal

**Figure 1:**
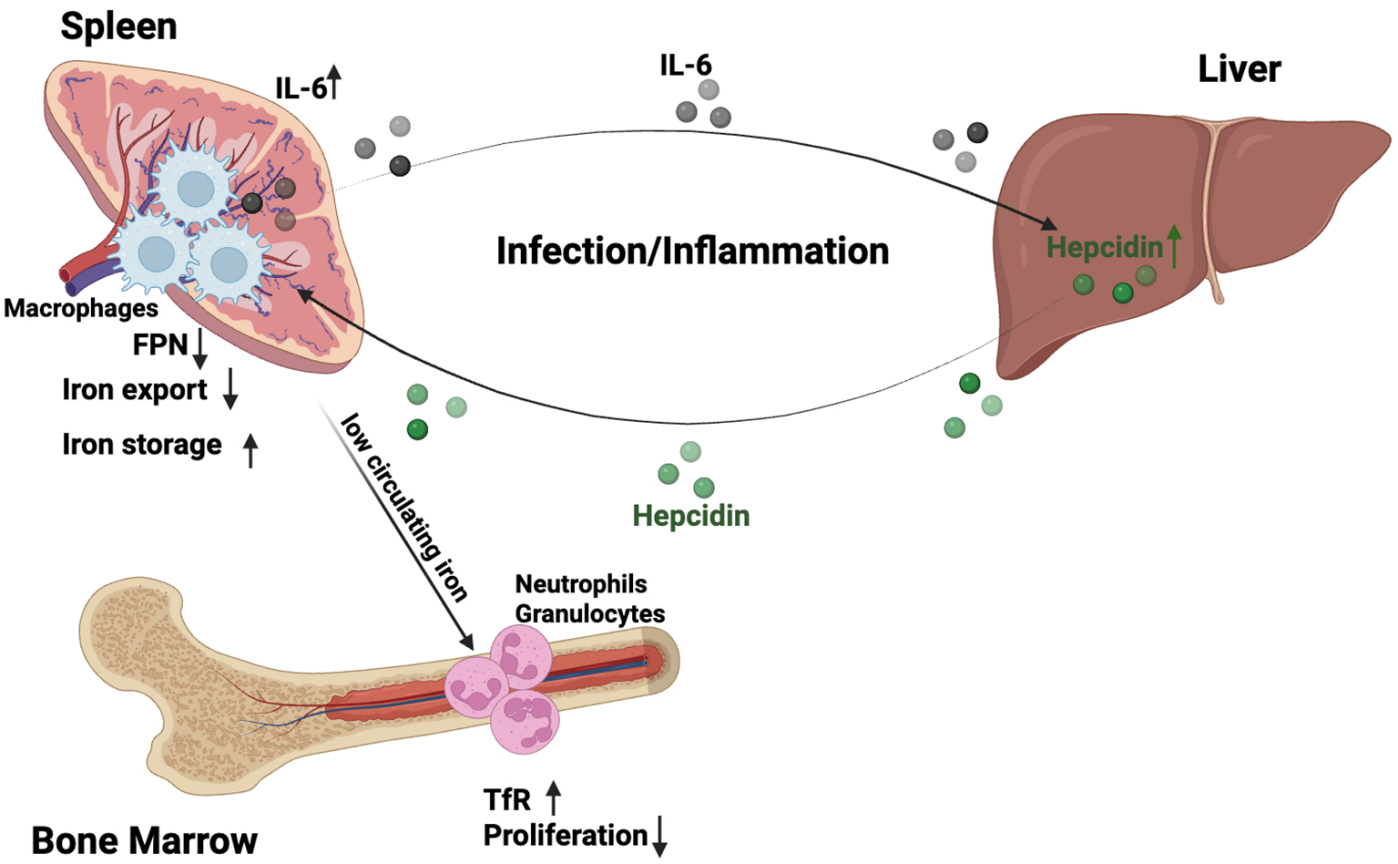
Systemic iron redistribution as an immunoregulatory signal during inflammation/infection. Inflammatory cues, prominently interleukin-6 (IL-6), induce hepatic hepcidin production, leading to ferroportin (FPN) internalization and reduced iron export into the circulation. This results in systemic hypoferremia characterized by low plasma iron, while total body iron remains largely compartmentalized in intracellular pools. Reduced circulating iron availability selectively constrains granulopoiesis by limiting iron-dependent proliferation and differentiation of neutrophil progenitors, which exhibit high transferrin receptor 1 (TfR1) expression and iron demand. In contrast, monocyte and macrophage production is relatively preserved under the same systemic conditions. Splenic and tissue-resident macrophages maintain cytokine output and antimicrobial activity, thereby supporting host defense while granulocyte supply is reduced. Figure was created with BioRender.

During infection and inflammation, systemic iron homeostasis is actively remodeled as an integral component of the host immune response. Inflammatory cues, most prominently interleukin-6, induce the expression of the master iron regulator hepcidin, resulting in internalization of the sole cellular iron exporter ferroportin (FPN) and reduced iron export into the circulation. In addition, cytokines, microbial products and acute phase proteins induce iron acquisition and retention in macrophages through hepcidin-independent pathways (6–8). These combined actions produce a state of hypoferremia characterized by low plasma iron concentrations, while total body iron remains largely buffered within intracellular ferritin- and heme-bound pools (9, 10) (**Figure 1**).

Experimental *in vivo* studies demonstrate that plasma iron availability directly influences immune cell production and function. Hepcidin-driven hypoferremia selectively constrains granulopoiesis by limiting iron-dependent proliferation and differentiation of neutrophil progenitors, which exhibit high transferrin receptor 1 (TfR1) expression and iron demand. In contrast, monocyte and macrophage compartments are relatively preserved under the same systemic conditions, maintaining cytokine production and antimicrobial activity (11).

Systemic iron signals are further interpreted at the cellular level through regulated intracellular iron handling. During intracellular bacterial infection, host immune signaling can locally override systemic hypoferremia. In macrophages infected with *Salmonella*, interferon-γ (IFN-γ) and nitric oxide-dependent activation of the redox-sensitive transcription factor nuclear factor erythroid 2-related factor 2 (NRF2) induces FPN expression, promoting iron export from infected cells despite low circulating iron. This response limits iron acquisition by intracellular bacteria while reinforcing pro-inflammatory cytokine signaling (12, 13).

The immunoregulatory consequences of iron redistribution extend beyond innate immunity to adaptive immune responses. Iron availability critically shapes lymphocyte activation, proliferation, and effector differentiation (14–16). *In vivo* models of dietary iron deficiency demonstrate impaired CD8^+^ T cell expansion and reduced effector cytokine production during viral infection and vaccination, despite preserved antigen recognition (17–19). In line with this concept, genetic defects in cell-intrinsic iron uptake, such as hypomorphic mutations in the transferrin receptor gene *TFRC*, cause combined immunodeficiency with defective lymphocyte development despite normal systemic iron parameters, underscoring the non-redundant role of iron-dependent signaling in immune competence (20, 21). Beyond T lymphocytes, iron availability also influences additional lymphoid populations. Iron-dependent epigenetic regulation has been shown to control B-cell proliferation and antibody responses (22), while recent work indicates that transferrin receptor-mediated iron uptake supports the metabolic fitness and effector function of innate lymphoid populations including ILC3 and NK cells (23, 24). Collectively, these observations highlight that myeloid and lymphoid cells differ fundamentally in how they sense and respond to iron: while myeloid cells primarily regulate iron availability as part of antimicrobial defense and inflammatory control, lymphoid cells depend on tightly regulated iron uptake to sustain proliferation, metabolic activation, and effector differentiation, rendering them particularly vulnerable to systemic iron restriction. A comprehensive overview of iron-dependent regulatory mechanisms across immune cell populations is provided elsewhere (5).

Importantly, systemic iron regulation sets physiological trade-offs. This is evident in infection models, where baseline iron status critically determines disease outcome. In murine *Salmonella* infection, dietary iron deficiency impairs antimicrobial immunity, whereas iron supplementation in anemic hosts worsens infection outcomes by increasing pathogen iron access and dysregulating anti-microbial immune responses (25). Sustained hypoferremia contributes to the development of anemia of inflammation and can compromise immune resilience under chronic inflammatory conditions (26). Recent work demonstrates that macrophage iron retention exerts lineage-specific effects on hematopoiesis, preferentially impairing erythropoiesis while preserving myeloid immune compartments (27).

Beyond adult hematopoiesis, hepcidin-dependent iron restriction shapes early developmental programs. Genetic deletion or pharmacological elevation of hepcidin disrupts embryonic iron homeostasis and erythroid differentiation *in vivo*, resulting in anemia, impaired progenitor maturation, and ferroptosis-associated stress responses during development (28, 29). Notably, bone marrow stromal cells have recently been identified as a local source of hepcidin in close spatial proximity to hematopoietic stem and progenitor cells, providing direct evidence that iron regulation operates within specialized immune niches rather than exclusively through systemic circulation (30).

Collectively, these findings establish systemic iron redistribution as an immunoregulatory mechanism that integrates inflammatory signaling with immune cell production, activation thresholds, and effector function. Rather than acting solely as a host defense strategy against pathogens, iron availability conveys contextual information that shapes immune behavior across innate and adaptive compartments, providing the conceptual framework for the cell- and organelle-specific mechanisms discussed in subsequent sections.

## 3 Tissue- and niche-specific iron handling: macrophage heterogeneity in the spleen

**Figure 2:**
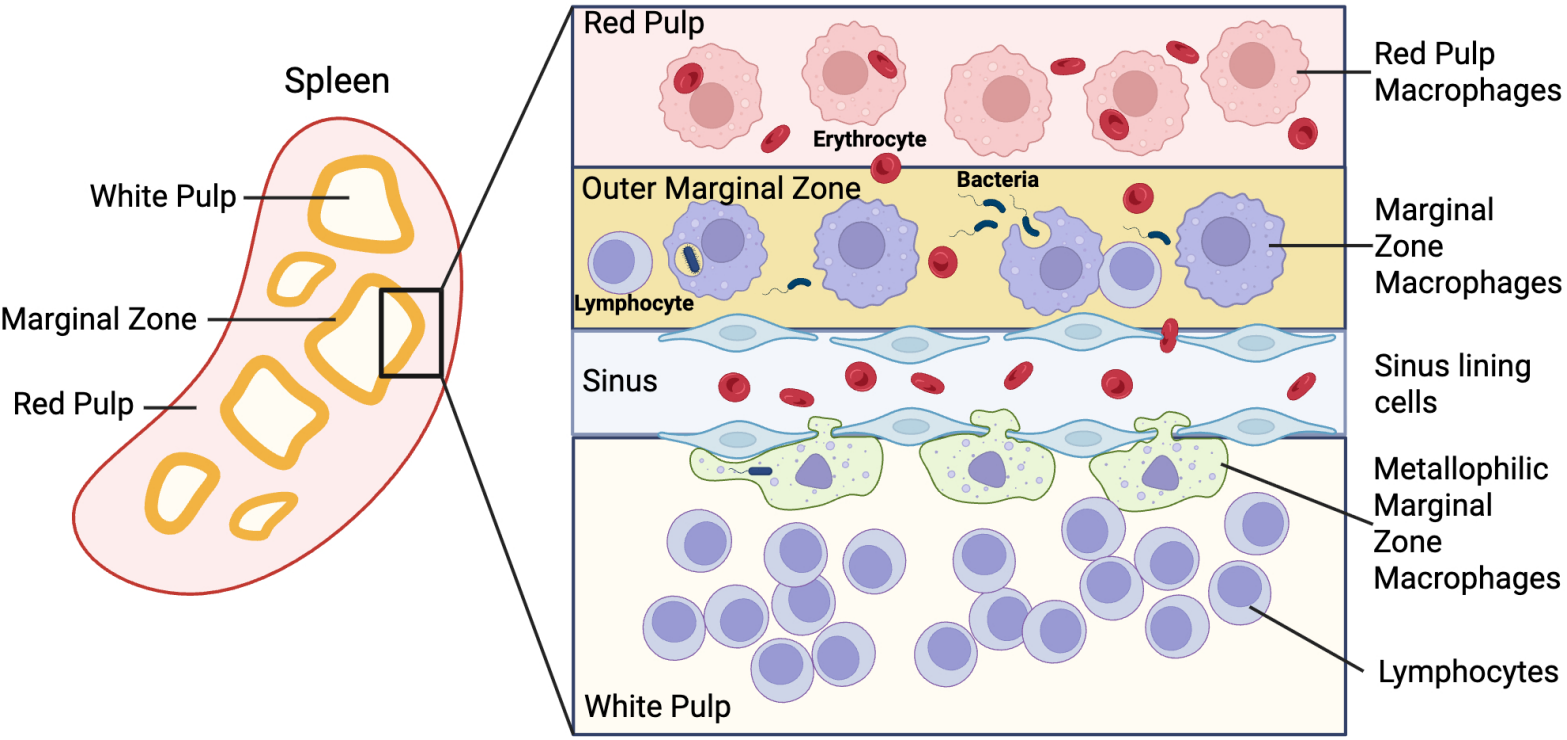
Spatial organization and functional specialization of splenic macrophage subsets. The spleen is organized into distinct anatomical niches that host specialized macrophage populations with non-redundant functions in iron handling and immune defense. Red pulp macrophages (RPMs) reside within the red pulp, where they clear senescent erythrocytes and support systemic iron recycling through erythrophagocytosis. Marginal zone macrophages (MZMs), positioned at the blood-splenic interface, rapidly capture blood-borne pathogens and particulate antigens, facilitating early innate immune defense and antigen relay to lymphocytes. Marginal metallophilic macrophages (MMMs) line the marginal sinus and contribute to immune surveillance and antigen handling at the interface between marginal zone and white pulp. This spatial segregation enables the spleen to simultaneously coordinate iron recycling, pathogen containment, and adaptive immune activation, providing a framework for niche-specific interpretation of systemic iron signals during infection and inflammation. Figure was created with BioRender.

The spleen contains a highly specialized macrophage network that is spatially organized across distinct anatomical compartments, enabling the parallel execution of iron recycling, immune surveillance, and antigen handling. Red pulp macrophages (RPMs) are positioned within the splenic cords and sinusoids, where they mediate the clearance of senescent erythrocytes and support systemic iron turnover. In contrast, macrophages of the marginal zone fulfill primarily immunological functions at the blood-lymphoid interface. Marginal zone macrophages (MZMs) capture blood-borne microbes and particulate antigens and facilitate their transfer to B cells, thereby supporting rapid humoral responses, whereas marginal metallophilic macrophages (MMMs) retain antigens at the marginal sinus and promote antigen delivery to the white pulp and T-cell areas. This spatial organization establishes a division of labor in which macrophage localization determines iron handling and immune function, creating splenic niches that interpret systemic inflammatory and iron-related signals in distinct ways (31, 32) (**Figure 2**).

Within this framework, splenic red pulp macrophages are central to systemic iron homeostasis, recycling approximately 90% of daily iron demand through erythrophagocytosis followed by heme oxygenase-1-dependent iron liberation and FPN-mediated export (1, 33). Recent work has refined this classical view by identifying regulatory nodes that tune RPM iron handling. Neudesin, a secreted MAPR family protein with a heme-binding domain, acts as a negative regulator of erythrophagocytosis. Loss of Neudesin accelerates erythrocyte clearance, depletes splenic iron stores, and delays recovery from anemia by impairing ERK1/2-dependent phagocytic signaling (34). These findings indicate that RPM iron fluxes are dynamically regulated rather than constitutively fixed.

RPM identity and function are specified by lineage-defining transcriptional programs. Spi-C and Notch2 signaling are required for RPM differentiation, and their disruption leads to iron accumulation and failure to support erythropoiesis during inflammatory stress (35). Functionally, RPMs display an iron-recycling phenotype characterized by high expression of heme oxygenase-1 (HO-1), FPN, and CD163, prioritizing iron export over inflammatory effector functions (33, 36). Despite uniform systemic inflammatory signals, splenic red pulp macrophages maintain FPN expression through local iron regulatory protein 2 (IRP2) dependent post-transcriptional regulation, preserving iron export capacity, whereas hepatic Kupffer cells rapidly internalize FPN to enforce liver-specific hypoferremia (37). During systemic *Salmonella* infection, splenic iron loss occurs despite expansion of RPMs and systemic hypoferremia, driven by IFN-γ inducible nitric oxide synthase (iNOS)-dependent FPN upregulation at the protein level (12, 38). This response uncouples local iron handling from classical inflammatory sequestration and illustrates the selective resilience of splenic iron-recycling macrophages under inflammatory stress (11).

Beyond RPMs, macrophage populations of the splenic marginal zone are specialized in immune surveillance and antigen relay. MZMs and MMMs rapidly capture blood-borne particles and pathogens and transfer antigens to B cells via SIGN-R1-dependent mechanisms and induce CD8+ mediated immune effector functions (39–42). In contrast to RPMs, marginal zone and marginal metallophilic macrophages are particularly sensitive to inflammatory stress and are frequently reduced during severe blood-borne infections, reflecting inflammation-driven remodeling of the splenic microenvironment (31). Although marginal zone macrophages are not directly involved in iron recycling, their rapid sensing of blood-borne pathogens and shaping of early inflammatory responses critically influence downstream iron-regulatory programs in the spleen. *In vivo* depletion studies further demonstrate functional non-redundancy among splenic macrophage subsets, with RPMs being indispensable for clearance of infected erythrocytes, whereas MZMs and MMMs predominantly support CD4^+^ T cell activation and cytokine production during blood-borne infection (43).

Together, these niche-specific macrophage programs define the tissue context in which iron-dependent immune regulation occurs. However, how these iron fluxes are sensed and translated into intracellular signaling decisions remains incompletely understood. At the subcellular level, lysosomes have recently emerged as important regulatory platforms that link iron trafficking with immune signaling and antimicrobial host defense.

## 4 Lysosomal iron as an integrative hub: linking iron trafficking to immune signaling

While tissue-specific iron handling explains how identical systemic cues generate divergent immune outcomes, the question remains how iron availability is sensed and translated into signaling decisions at the subcellular level.

**Figure 3:**
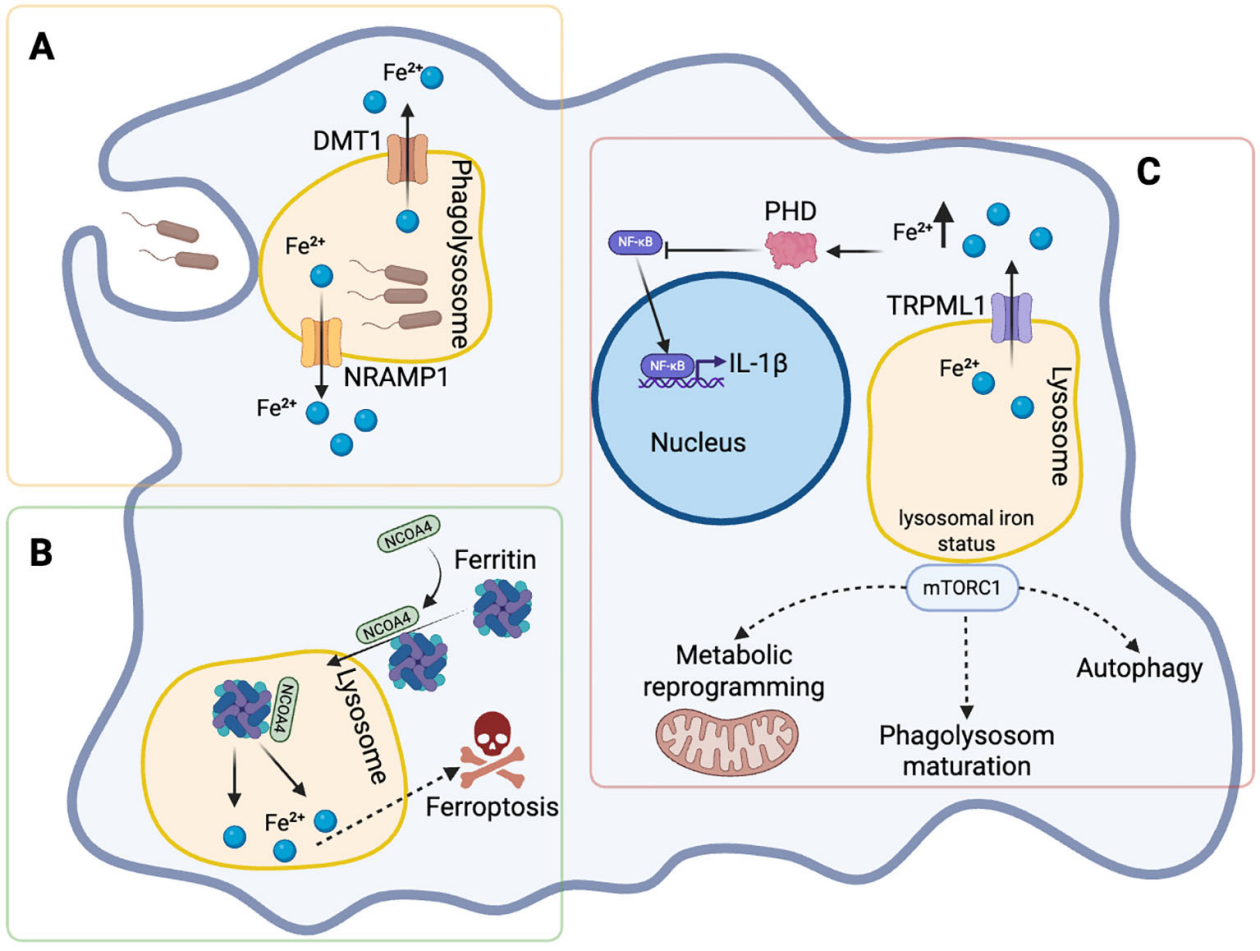
Lysosomal iron integrates antimicrobial defense, inflammatory feedback, and metabolic decision-making in macrophages. **(A)** In pathogen-containing phagolysosomes, iron availability is restricted through the coordinated action of the divalent metal transporters NRAMP1 and DMT1, which export Fe²^+^ from the phagolysosomal lumen to the cytosol, thereby limiting iron access to intracellular pathogens as part of nutritional immunity. **(B)** Ferritin-bound iron is mobilized through NCOA4-mediated ferritinophagy, resulting in lysosomal iron release and dynamic adjustment of intracellular iron pools. Under conditions of dysregulated iron handling, excessive iron liberation can predispose cells to iron-dependent cytotoxicity, exemplified by ferroptosis. **(C)** Beyond antimicrobial iron restriction, lysosomal iron also functions as a signaling intermediate. In response to inflammatory stress, lysosomal Fe²^+^ is released into the cytosol via the lysosomal cation channel TRPML1, leading to activation of prolyl hydroxylases (PHDs) and subsequent attenuation of nuclear factor κB- (NF-κB) dependent interleukin-1 β (IL-1β) transcription, thereby calibrating inflammatory output. In parallel, lysosomal iron status intersects with mechanistic target of rapamycin complex 1 (mTORC1) activity at the lysosomal surface, influencing context-dependent cellular programs including metabolic reprogramming, autophagy, and phagolysosome maturation. Collectively, these pathways position the lysosome as an integrative platform linking iron trafficking to immune, metabolic, and antimicrobial responses. Figure was created with BioRender.

Lysosomes have recently emerged as a crucial integrative hub that links iron trafficking and metabolism to immune signaling and antimicrobial defense. Lysosomes are central organelles in iron homeostasis by coordinating the degradation of TfR1 (44–46), mediating the degradation of ferritin through ferritinophagy (47, 48) as well as by promoting the autophagocytic degradation of mitochondria (49) and erythrocyte turnover for iron recycling (50). In parallel, lysosomes play a crucial role in infection management by fusing with phagosomes (51). Since pathogen survival and replication go hand in hand with iron availability, lysosomes need to tightly control iron access to intruders (52) (**Figure 3**).

### 4.1 Phagolysosomes, Nramp1 and nutritional immunity

Phagocytic cells like macrophages engulf pathogens into the so-called phagosome. In order to exterminate the engulfed microbe, the phagosome needs to mature into the phagolysosome by fusing with the lysosome, where the acidic and oxidative conditions promote the degradation of the phagocytosed content (51).

Natural resistance-associated macrophage protein 1 (Nramp1) is a divalent metal transporter localized to late endosomes, lysosomes, and phagosomes/phagolysosomes. It has long been recognized as a key determinant of resistance to intracellular pathogens such as *Salmonella* and *Mycobacterium tuberculosis* by modulating intraphagosomal metal availability and antimicrobial effector pathways (53).

Subsequent studies established that Nramp1 exports iron from the phagosome into the cytosol and subsequently reduces intracellular iron pools by enforcing FPN-1 mediated iron export, which restricts the iron availability for intracellular pathogens within infected macrophages (54). This subcellular iron trafficking represents a core mechanism of nutritional immunity at the phagolysosomal interface and illustrates how subcellular iron trafficking directly constrains pathogen replication (53, 55). Pathogens such as *Salmonella Typhimurium* or *Leishmania major* have evolved strategies to counteract this defense by destabilizing Nramp1 and increasing iron availability within the phagolysosome, thereby promoting intracellular survival (56, 57).

Together, there is compelling evidence that phagolysosomes export iron to the cytosol via the Nramp1 transporter, thereby limiting iron access to intracellular pathogens such as *Salmonella*, *Leishmania*, and *Mycobacterium tuberculosis*, while concomitantly reshaping lysosomal and cytosolic iron availability in the host cell (4) (**Figure 3A**). In turn, pathogens have evolved mechanisms to circumvent this iron restriction by downregulating Nramp1 expression or getting access to alternative iron sources (57, 58).

### 4.2 Lysosomes and DMT-1-iron handling upon inflammatory signals

During *Salmonella* infection, macrophage iron transport pathways directly shape antimicrobial defense. Macrophage-derived lipocalin-2 (LCN2) limits bacterial iron acquisition by sequestering catecholate siderophores, thereby restricting iron availability for bacteria (8). Disruption of upstream iron transport via DMT1 deficiency impairs LCN2 induction and elevates intracellular iron pools, exacerbating bacterial replication and increasing susceptibility to systemic infection (59).

Following transferrin-TfR1-mediated iron uptake and endosomal acidification, ferrous iron is released into the endolysosomal lumen and subsequently transported to the cytosol primarily via divalent metal transporter 1 (DMT1). This transport step is tightly regulated by oxidoreductases (48). DMT1 is also expressed at the plasma membrane, where it mediates cellular uptake of divalent cations (46, 60). By governing iron efflux from endolysosomal compartments, DMT1 influences the balance between vesicular iron retention and cytosolic iron availability.

DMT1 expression and activity are responsive to inflammatory cues (6). In non-infectious inflammatory contexts, including neurodegenerative and degenerative diseases such as Alzheimer’s disease, Parkinson’s disease, and osteoarthritis, pro-inflammatory cytokines and innate immune stimuli have been shown to induce DMT1 expression, promoting cytosolic iron accumulation and cellular stress. In neurons, inflammation activates nuclear factor κB- (NF-κB) dependent transcription of the endolysosomal DMT1 isoform, resulting in increased iron export from endosomes into the cytosol (61). Similarly, in chondrocytes, IL-1β induces both transcriptional and translational upregulation of DMT1, enhancing cytosolic iron influx (62).

In the context of infection, macrophage-intrinsic DMT1 also contributes to iron-dependent host defense. Genetic deletion of DMT1 in macrophages increases iron availability for *Salmonella*, in part by reducing LCN2 induction, thereby promoting bacterial replication and increasing susceptibility to systemic infection (59). Given that DMT1 can localize to phagosomal membranes in macrophages (63), loss of DMT1 may additionally impair iron export from pathogen-containing vacuoles, leading to iron retention within the intracellular niche and altered inflammatory responses.

### 4.3 Lysosomes and ferritinophagy

Ferritin expression is regulated by inflammatory cytokines to limit pathogen access to iron. Ferritinophagy enables context-dependent adjustment of intracellular iron availability in response to metabolic and inflammatory demands. The selective degradation of ferritin in lysosomes releases iron and increases cellular iron availability, thereby modulating the size of labile iron pools in response to inflammatory stimuli (64, 65). This autophagic degradation of ferritin can be exploited by pathogens to acquire iron. For example, *Ehrlichia chaffeensis* induces ferritinophagy through a type IV-secreted effector that binds ferritin, increasing the labile iron pool and promoting intracellular proliferation (66).

Beyond facilitating iron acquisition, excessive or dysregulated ferritinophagy can convert lysosomal iron release from a regulatory signal into a trigger of iron-dependent cytotoxic programs, including ferroptosis. In viral infections such as pseudorabies infection, coordinated induction of iron uptake pathways and ferritinophagy mediated by nuclear receptor coactivator 4 (NCOA4) promotes ferroptotic cell death. This process facilitates viral replication and illustrates how pathogens exploit lysosomal iron release not only for metabolic gain but also to manipulate host cell fate (67) (**Figure 3B**).

### 4.4 Lysosomal iron mediates cellular immune response

Lysosomal derived iron functions as an active regulator of immune signaling in monocytes and macrophages by reducing the formation of cytokines such as TNF-α, IL-1β or IL-6 in response to inflammatory stimuli or IFN-γ (68–70). While iron appears to affect IFN-γ driven macrophage activation by interfering with signaling processes and modulation of mRNA stability (71), lysosomal Fe²^+^ can induce reactive oxygen species generation which then activate the lysosomal cation channel TRPML1, triggering Fe²^+^ release into the cytosol. This lysosomal iron flux precedes transcriptional feedback mechanisms that constrain inflammatory output. An increase in cytosolic Fe²^+^ activates prolyl hydroxylases, which repress NF-κB activity and thereby suppress *IL1B* transcription, ultimately attenuating proinflammatory cytokine production (72) (**Figure 3C**).

Together, these findings establish lysosomal iron trafficking as a rheostat that calibrates macrophage inflammatory responses by coupling iron flux to NF-κB-dependent transcriptional control. Beyond inflammatory feedback regulation, lysosomal iron availability also intersects with mTOR-dependent metabolic and autophagic decision-making during infection.

### 4.5 mTOR in infection and the effect of iron

Mechanistic target of rapamycin complex 1 (mTORC1) is a central integrator of cellular nutrient and energy status that is activated at the lysosomal surface under nutrient-replete conditions and suppressed during energy stress (73, 74). Current evidence suggests that iron influences mTORC1 activity indirectly through metabolic and stress-sensing pathways. Iron availability intersects with mTORC1 signaling by shaping lysosomal function, membrane composition, and organelle integrity. Through this indirect coupling, mTORC1 translates infection- and inflammation-associated changes in lysosomal iron handling into coordinated metabolic reprogramming, autophagic control, lysosomal biogenesis, and cytokine production. Iron deficiency can activate AMPK- and hypoxia-associated signaling programs that converge on mTORC1 repression, whereas iron repletion restores mTOR signaling by normalizing cellular metabolic state (75). Consistent with this concept, recent work identified an iron-dependent histone demethylase (KDM3B) that functions as an upstream iron sensor controlling the expression of key components of the mTORC1 pathway, thereby linking cellular iron availability to anabolic signaling. Notably, this mechanism has so far mainly been characterized in non-immune cell systems (76). Of note, iron availability is centrally involved in the control of metabolic processes via its function as a cofactor for multiple metabolic enzymes but also by regulating mitochondrial aconitase translation via IRPs and thus TCA cycle activity and oxidative phosphorylation (1, 77). Accordingly, alterations in cellular and systemic iron homeostasis affect metabolite composition including the balance of pyruvate/lactate, glucose consumption and fatty acid formation (78, 79). Importantly, mTOR also impacts on iron homeostasis by regulating TfR-dependent iron uptake via modulation of tristetraprolin availability (80).

#### 4.5.1 Iron modulates mTOR-driven metabolic reprogramming during infection

It is well established that mTOR-dependent metabolic reprogramming occurs in immune cells upon infection (81). In macrophages, activation of mTORC1 promotes a Warburg-like metabolic state characterized by enhanced glycolysis and reduced TCA cycle activity, thereby supporting the energetic and biosynthetic demands of cytokine production and immune cell activation (82). Remarkably, iron availability modulates this mTORC1-driven metabolic program during intracellular infection. In macrophages infected with *Salmonella*, mTOR activation supports a glycolytic metabolic profile with reduced TCA cycle activity. Iron supplementation partially shifts this metabolic configuration by increasing the expression and activity of TCA cycle enzymes and promoting a partial restoration of aerobic metabolism. Functionally, inhibition of mTOR impairs bacterial control, an effect that is further exacerbated by iron supplementation, indicating that iron availability reshapes mTOR-dependent metabolic states in ways that can favor pathogen persistence (83). Of interest, NLRP6 controls iron access for *Salmonella* by regulating NRF2-mediated ferroportin expression via mTOR function (84). In addition, deletion of IRPs in macrophages results in reduced control of *Salmonella* infection due to reduced formation of the antimicrobial peptide LCN2, uncontrolled iron access to bacteria, and presumably an altered intracellular metabolite composition (8).

Together, these findings indicate that iron does not merely serve as a nutrient for intracellular pathogens but acts as an immunometabolic signal that reprograms mTORC1-dependent metabolic decisions in infected macrophages, thereby shaping the balance between glycolytic and oxidative metabolism during infection.

#### 4.5.2 mTOR-induced phagolysosome maturation is influenced by iron

mTORC1 plays a central role in regulating phagolysosome maturation and acidification during infection, thereby shaping pathogen clearance. In macrophages infected with *Vibrio vulnificus*, pharmacological inhibition of mTORC1 impairs bacteria-induced phagosomal acidification and phagolysosome formation, resulting in increased bacterial survival (52). In contrast, *Salmonella* actively exploits mTORC1 signaling to promote its intracellular persistence. *Salmonella* induces cholesterol accumulation at the *Salmonella*-containing vacuole, which is required for mTOR recruitment and activation, leading to suppression of autophagy and impaired pathogen clearance (85). Iron availability further intersects with phagolysosome maturation pathways. In macrophages infected with *Mycobacterium avium*, elevated intracellular iron levels impair phagolysosome maturation and promote bacterial survival, whereas iron chelation restores phagolysosomal maturation and enhances pathogen degradation (86). Mechanistically, iron homeostasis influences lysosomal function through an IRP2-transcription factor EB (TFEB) axis. IRP2 supports lysosomal biogenesis and acidification by promoting TFEB nuclear translocation and transcription of lysosomal genes. Loss of IRP2 compromises these processes, leading to reduced lysosome formation (87). As IRP2 stability is negatively regulated by iron, elevated intracellular iron levels are predicted to suppress IRP2-TFEB-dependent lysosomal biogenesis.

Beyond regulating lysosomal iron trafficking and metabolic signaling pathways such as mTORC1, fluctuations in cellular iron availability also influence mitochondrial metabolism (2, 3, 88). Because mitochondria represent major sites of iron utilization, numerous enzymes of the TCA cycle and oxidative phosphorylation depend on iron-containing cofactors. Accordingly, cellular iron availability links lysosomal iron handling and metabolic signaling to mitochondrial function during infection and inflammation.

## 5 Mitochondrial iron as an immunometabolic checkpoint

Mitochondria are central organelles in immune cells where iron availability directly shapes metabolic configuration, redox balance and oxidative phosphorylation, with immediate consequences for inflammatory activation (2, 89). Experimental manipulation of iron availability in primary immune cells demonstrates that iron status controls mitochondrial respiration and the balance between oxidative phosphorylation and glycolysis, thereby tuning inflammatory output (90).

**Figure 4:**
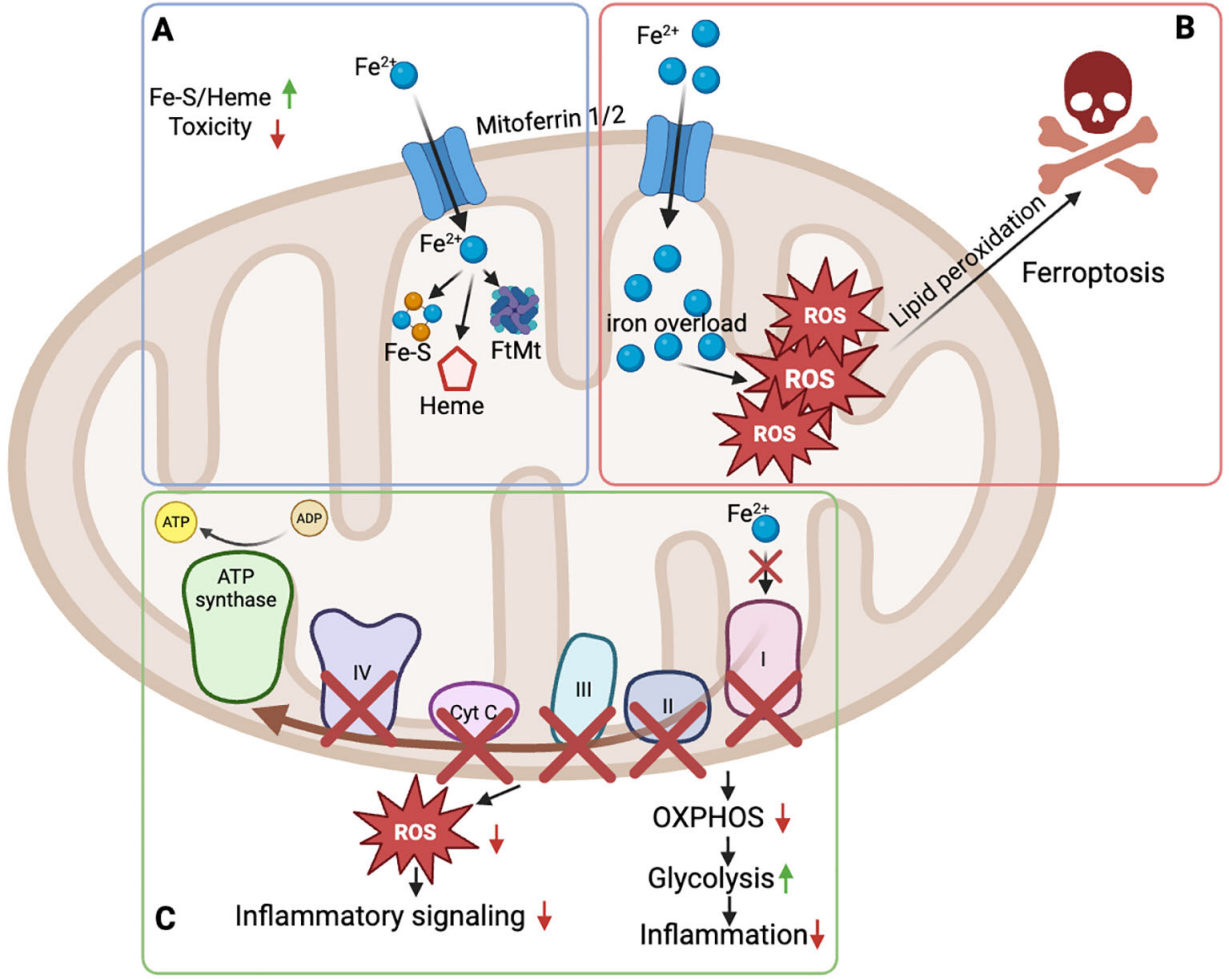
Mitochondrial iron availability defines immunometabolic thresholds in immune cells. Mitochondrial iron handling calibrates metabolic capacity, redox balance, and inflammatory signaling in immune cells through distinct functional states. **(A)** Balanced mitochondrial iron utilization. Import of ferrous iron (Fe²^+^) via mitoferrin-1/2 supports iron-sulfur cluster (Fe-S) and heme biosynthesis, enabling proper function of electron transport chain (ETC) complexes and ATP synthase. Iron buffering by mitochondrial ferritin (FtMt) limits labile iron and prevents iron-driven toxicity, maintaining oxidative phosphorylation (OXPHOS) with low reactive oxygen species (ROS) production. **(B)** Mitochondrial iron overload. Excessive mitochondrial Fe²^+^ accumulation overwhelms buffering capacity, leading to elevated mitochondrial ROS generation, lipid peroxidation, and ferroptotic cell death. **(C)** Functional mitochondrial iron limitation. Insufficient mitochondrial iron impairs Fe-S and heme-dependent ETC complexes (I-IV), reducing OXPHOS and ATP production, promoting a compensatory shift toward glycolysis, and attenuating redox-dependent inflammatory signaling. Figure was created with BioRender.

Beyond supporting oxidative metabolism, mitochondrial iron availability determines the functionality of iron-sulfur cluster (ISC) containing respiratory complexes and heme-dependent enzymes, positioning mitochondrial iron handling as a key determinant of bioenergetic capacity and inflammatory competence (91, 92). In this context, mitochondrial iron emerges as an immunometabolic checkpoint that translates iron availability into metabolic and inflammatory responses during immune activation (**Figure 4**).

### 5.1 Mitochondrial iron handling and compartmentalization

Mitochondrial iron handling relies on strict compartmentalization to enable ISC and heme biosynthesis while preventing iron-induced toxicity (2, 91) (**Figure 4A**).

Perturbations in mitochondrial iron availability primarily compromise ISC-dependent respiratory functions, resulting in constrained oxidative metabolism rather than global mitochondrial failure. In immune cells, these constraints reshape metabolic states associated with inflammatory activation (90). Dysregulated mitochondrial iron handling further predisposes immune cells to metabolic collapse and iron-dependent cell death under activation stress, underscoring the necessity of tight compartmentalization of mitochondrial iron pools (93).

### 5.2 Iron-dependent control of mitochondrial metabolism

As a consequence, limited mitochondrial iron availability reshapes immune cell metabolic programming and lowers the threshold for inflammatory dysfunction (**Figure 4C**). In primary human macrophages, acute iron deprivation suppresses oxidative phosphorylation, reduces TCA cycle activity, and induces a compensatory shift toward glycolysis, establishing iron availability as a determinant of mitochondrial metabolic configuration rather than a passive cofactor supply (90).

Evidence from human disease, specifically in neuro-degenerative diseases, further supports this concept (94, 95). In patients with restless legs syndrome, reduced expression of mitochondrial iron genes is associated with impaired aconitase activity and diminished respiratory capacity, demonstrating that limited mitochondrial iron availability translates into functional defects of oxidative metabolism in primary human cells (96). Accordingly, iron chelator therapy in patients with early onset of Parkinson’s disease resulted in worsening of motoric function (97). Notably, systemic iron deficiency does not uniformly impair mitochondrial function across tissues, as peripheral blood mononuclear cells display distinct mitochondrial adaptations compared with non-immune organs, highlighting pronounced cell-type specificity of iron-dependent mitochondrial responses (98).

Consistently, dysregulated mitochondrial iron handling in naïve CD4^+^ T cells disrupts the metabolic transition required for immune activation, resulting in impaired oxidative metabolism and reduced functional competence (93). Collectively, these studies establish mitochondrial iron availability as a central regulator of cell metabolism that shapes inflammatory responses during activation.

### 5.3 Mitochondrial iron, mtROS, and inflammatory signaling

Mitochondria-derived reactive oxygen species (mtROS) act as central signaling intermediates that couple mitochondrial metabolic state to innate immune activation. Seminal work demonstrated that mitochondrial integrity and mtROS generation are required for NLRP3 inflammasome activation, positioning mitochondria as active regulators of IL-1β maturation (99). Subsequent studies showed that mitochondrial stress triggers lysosomal membrane permeabilization in an NLRP3-dependent manner, thereby coupling mitochondrial state not only to cytokine release but also to inflammatory cell fate decisions (100).

Within this signaling framework, mitochondrial iron availability modulates the redox capacity of mitochondria rather than directly initiating inflammatory pathways. By shaping respiratory chain efficiency and electron flux, mitochondrial iron content determines the amplitude of mtROS generation under activating conditions, thereby influencing redox-sensitive inflammatory signaling cascades. Importantly, this positions mitochondrial iron as a permissive factor that calibrates mtROS-dependent signaling responses (101).

In parallel, inflammatory cues actively remodel mitochondrial iron buffering to restrain excessive redox stress. Pro-inflammatory stimulation induces mitochondrial ferritin (FtMt) expression, which sequesters iron within mitochondria and limits iron-driven oxidative damage, thereby modulating redox-sensitive inflammatory signaling in neural cells and inflammatory disease models (102).

Consistent with this model, experimental iron loading amplifies inflammatory signaling by perturbing mitochondrial redox homeostasis. Elevated intracellular iron increases mitochondrial superoxide production and potentiates lipopolysaccharide-induced cytokine release in primary macrophages, effects that are reversed by mitochondria-targeted antioxidants (101). Together, these findings indicate that mitochondrial iron availability calibrates mtROS-dependent inflammatory signaling by tuning mitochondrial redox capacity, rather than serving as a direct trigger of inflammasome activation.

### 5.4 Mitochondrial iron and immune cell fate decisions

Beyond metabolic control, mitochondrial iron availability influences immune cell fate decisions under conditions of activation and stress. Balanced mitochondrial iron handling supports bioenergetic capacity, immune cell survival, and functional responses, whereas dysregulated iron accumulation predisposes cells to iron-dependent cell death pathways (103). Pathological activation of ferritinophagy represents one mechanism by which mitochondrial and cellular iron buffering collapses during severe inflammation. In a murine model of sepsis-induced cardiac injury, lipopolysaccharide exposure triggered NCOA4-dependent ferritin degradation, excessive liberation of labile Fe²^+^, mitochondrial dysfunction, lipid peroxidation, and ferroptotic cell death. Genetic or pharmacological inhibition of ferritinophagy restored iron sequestration, preserved mitochondrial integrity, and attenuated inflammatory tissue damage (104) (**Figure 4B**).

Mechanistic evidence further demonstrates that ferritinophagy-driven iron release can directly fuel mitochondrial iron overload beyond infectious settings. In cardiomyocytes, NCOA4-mediated ferritinophagy increased mitochondrial iron accumulation via the mitochondrial iron transporter sideroflexin-1 (SFXN1), resulting in elevated mitochondrial reactive oxygen species and pathological cellular remodeling. Inhibition of ferritinophagy or mitochondrial iron import preserved mitochondrial function and prevented iron-dependent dysfunction, establishing a direct functional link between lysosomal ferritin degradation and mitochondrial iron toxicity (105).

In immune cells, recent evidence shows that disruption of mitochondrial iron homeostasis in naïve CD4^+^ T cells impairs the metabolic transition required for activation and increases susceptibility to iron-dependent cell death. Impaired heme export as well as reduced anti-oxidant capacities cause cellular and mitochondrial iron overload, defective respiratory adaptation, and loss of functional competence, defining a threshold between immune activation and metabolic failure (93, 106).

Iron-dependent cell death pathways such as ferroptosis represent extreme endpoints of this dysregulation. Ferroptosis is characterized by iron-dependent lipid peroxidation and mitochondrial dysfunction and reflects a breakdown of iron-buffering and redox control mechanisms rather than a physiological immune strategy (107, 108).

Collectively, these findings establish mitochondrial iron as a central immunometabolic checkpoint in immune regulation. Tightly controlled iron utilization sustains metabolic fitness, redox balance, and immune cell function, whereas dysregulated iron accumulation precipitates pathological cell fate decisions, including iron-dependent cell death, during inflammation and infection. Importantly, the metabolic and inflammatory consequences of host iron regulation are not confined to immune cells alone. From the pathogen perspective, host-driven iron redistribution constitutes a dynamic environmental signal that shapes microbial physiology, virulence, and persistence during infection.

Collectively, these findings illustrate how host cells translate fluctuations in iron availability into metabolic and inflammatory responses through mitochondrial iron-dependent pathways. However, iron availability during infection is not only interpreted by host immune cells. Pathogens themselves actively sense changes in environmental iron availability and adapt their metabolic programs and virulence strategies accordingly.

## 6 The pathogen perspective: bacterial iron acquisition in the context of host iron signaling

From the pathogen perspective, iron availability represents not merely a nutritional constraint imposed by the host but a dynamic environmental signal that shapes bacterial physiology during infection. Host-driven iron sequestration alters both the concentration and chemical form of accessible iron, providing contextual cues that bacteria integrate into regulatory programs controlling metabolism, stress resistance, virulence, and persistence. Major host and bacterial systems controlling iron acquisition and intracellular iron handling are summarized in **Table 1** and have been comprehensively reviewed elsewhere (109–111).

### 6.1 Host-induced iron limitation as a signal for pathogens

**Figure 5:**
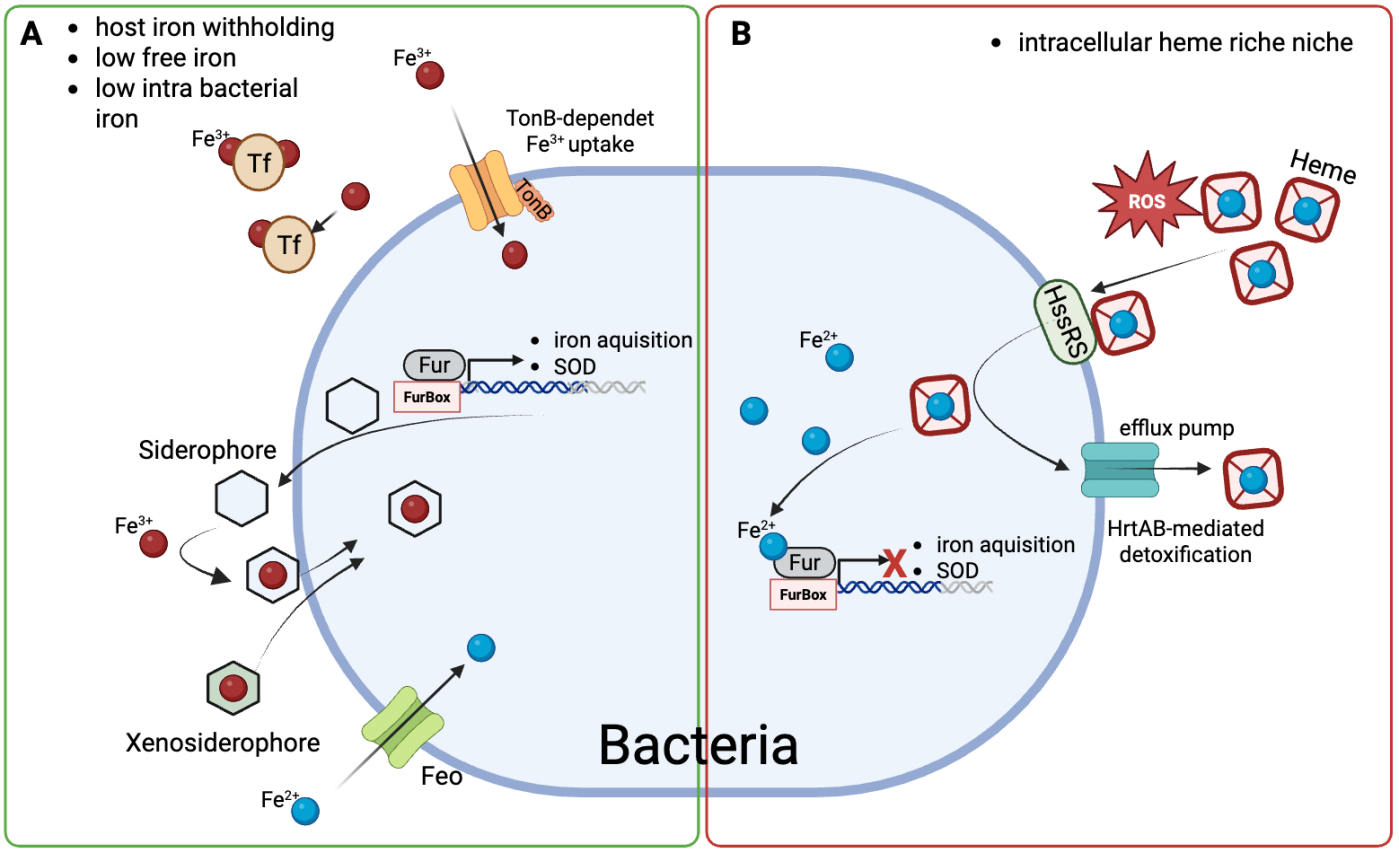
Bacterial interpretation of host-derived iron signals under iron-restricted and heme-rich conditions. The schematic bacterium shown represents a conceptual model rather than a specific Gram-positive or Gram-negative organism and illustrates conserved principles of bacterial iron sensing and adaptation to host-defined iron environments. **(A)** Iron-restricted host environment. During infection-associated iron withholding, freely available iron is scarce and largely protein-bound (transferrin, Tf). Under these conditions, intracellular iron levels are low and the ferric uptake regulator Fur predominantly exists in its iron-free (apo-Fur) form. This state promotes activation of iron acquisition pathways, including TonB-dependent uptake of ferric iron (Fe³^+^) and siderophores, as well as ferrous iron (Fe²^+^) uptake via Feo systems when available. **(B)** Heme-rich intracellular niche. In intracellular or erythrocyte-associated environments, bacteria encounter fluctuating iron conditions dominated by host-derived heme. Excess heme and iron impose oxidative and membrane stress. In this setting, Fur is predominantly iron-bound (holo-Fur), contributing to repression of iron uptake systems. Simultaneously, specialized heme-sensing pathways such as the HssRS two-component system detect heme-induced toxicity and induce protective responses, most prominently HrtAB-mediated efflux and detoxification. This regulatory configuration prioritizes stress protection while preventing further iron overload. Together, these scenarios illustrate that bacterial iron homeostasis is regulated by iron form, localization, and toxicity, enabling pathogens to dynamically adapt metabolism, virulence, and persistence strategies to host-imposed iron states. Abbreviations: Tf, transferrin; Fe²^+^, ferrous iron; Fe³^+^, ferric iron; Feo, ferrous iron transport system; HssRS, heme-sensing two-component regulatory system; HrtAB, heme-regulated transporter A/B. Figure was created with BioRender.

During infection and inflammation, host responses such as cytokine and hepcidin-driven hypoferremia and sequestration of extracellular iron drastically reduce freely available iron (112, 113). Recent *in vivo* work demonstrates that extracellular pathogens respond to this iron-restricted environment not simply with impaired growth, but by activating coordinated transcriptional programs linked to virulence and immune evasion (114). These observations indicate that immunity-driven restriction of host iron availability for invading pathogens results in adaptive responses of microbes to satisfy their metabolic needs (**Figure 5A**).

### 6.2 Bacterial iron sensing and regulatory circuits

Bacterial interpretation of host iron states is mediated through iron-responsive regulatory systems that couple intracellular iron levels to global gene expression. The ferric uptake regulator Fur represents a central integrative node, with genome-wide analyses revealing that Fur-controlled regulons extend far beyond iron acquisition to include oxidative stress resistance, metabolic flexibility, and virulence-associated pathways (115, 116) (**Figure 5A**).

Recent work demonstrates that iron limitation can act as a regulatory cue that promotes microbial lifestyle switching rather than causing growth arrest. In *Pseudomonas aeruginosa*, iron availability governs biofilm formation and persistence-associated gene expression, illustrating that bacterial responses to iron restriction are context-dependent and embedded in broader behavioral programs (117).

In addition to Fur, pathogens employ specialized sensory systems that respond to heme availability and heme-induced toxicity. In Gram-positive bacteria, the HssRS two-component system detects excess heme and induces protective responses such as HrtAB-mediated detoxification/efflux, balancing heme utilization and toxicity (118, 119).

### 6.3 Adapting iron acquisition strategies to host iron states

Bacterial iron acquisition strategies are dynamically adjusted to the host iron landscape. Under conditions dominated by transferrin-bound iron, pathogens preferentially engage TonB-dependent receptors and siderophore-mediated uptake systems to extract iron from host proteins (116). Rather than expressing all uptake pathways simultaneously, pathogens hierarchically deploy these systems according to iron chemistry and availability.

When extracellular iron becomes severely restricted, some bacteria shift towards utilization of xenosiderophores produced by competing microbes or activate ferrous iron transport systems such as the FeoABC transporter, which enable uptake of ferrous iron (Fe²^+^) under conditions where ferric iron is limited (116, 120, 121). These examples illustrate that bacterial iron acquisition is governed by regulatory decisions that interpret host iron states, rather than constitutive nutrient scavenging.

### 6.4 Intracellular and heme-rich niches: interpreting host-derived iron signals

Pathogens that encounter intracellular or heme-rich host environments are exposed to iron conditions that differ fundamentally from extracellular iron limitation. Within host cells or at sites of erythrocyte turnover, iron availability is shaped by host-controlled heme fluxes and detoxification mechanisms, generating fluctuating iron and heme landscapes rather than uniform iron starvation (2).

In this context, iron- and heme-responsive sensory systems enable pathogens to balance nutrient acquisition against toxicity (**Figure 5B**). In Gram-positive bacteria, the HssRS two-component system detects excess heme and induces protective responses, most prominently HrtAB-mediated efflux, thereby limiting heme-mediated toxicity while preserving the ability to exploit heme as an iron source (119). Recent mechanistic work has demonstrated that membrane-associated heme directly activates the sensor kinase HssS, establishing heme toxicity itself as the primary signal driving HssRS-dependent transcriptional responses (118) (**Figure 5B**).

Thus host-derived iron species, particularly heme, function as contextual signals that shape bacterial regulatory programs. Rather than responding solely to iron scarcity, pathogens integrate information about iron form, localization, and toxicity to fine-tune virulence-associated and stress-protection pathways, a principle increasingly recognized as central to pathogen fitness during infection (122).

### 6.5 Consequences for virulence, persistence, and therapeutic response

By integrating host-derived iron signals, pathogens dynamically modulate virulence factor expression, metabolic plasticity, and persistence strategies. Iron-restricted conditions promote biofilm formation, stress resistance, and entry into non-replicative states associated with antibiotic tolerance and chronic infection (114, 117, 123). Recent in-host evolutionary analyses further demonstrate that iron-dependent metabolic constraints can actively drive stable persistence phenotypes. In *Staphylococcus aureus*, a frameshift mutation in the iron-sulfur cluster (ISC) biogenesis gene *sufB* disrupted tricarboxylic acid cycle activity and electron transport, resulting in reduced ATP and reactive oxygen species production. This metabolic reprogramming promoted the formation of stable small colony variants with enhanced tolerance to antibiotics and neutrophil-mediated killing, leading to persistent inflammation *in vivo*. Restoration of *sufB* function reversed these phenotypes, establishing ISC deficiency as a causal determinant of persistence rather than a secondary consequence of host-imposed iron limitation (124).

Conversely, dysregulated iron acquisition can sensitize bacteria to oxidative stress and immune clearance, revealing vulnerabilities that may be therapeutically exploitable (116, 125). Collectively, these findings position bacterial iron acquisition as a signal-responsive system that interprets host iron states to guide pathogen behavior during infection. This perspective highlights the potential of targeting iron-dependent regulatory pathways, rather than iron availability alone, for antimicrobial intervention.

## 7 Conclusion and outlook

A unifying insight emerging from recent work is that inflammatory iron redistribution should not be understood solely as a host strategy to deprive pathogens of a limiting nutrient, but as a regulatory signal that actively shapes immune metabolism, differentiation, and effector capacity. Hepcidin-driven hypoferremia is often treated as a uniform systemic state; however, the immune consequences of iron restriction are highly non-uniform and depend on how systemic cues are interpreted across tissues, cell types, and subcellular compartments. This framework helps reconcile why inflammatory iron sequestration can enhance resistance to certain pathogens while simultaneously impairing hematopoiesis or adaptive immune responses in other contexts.

At the systemic level, inflammatory iron restriction does not globally suppress immune function but biases immune output. *In vivo* studies demonstrate that hepcidin-mediated hypoferremia selectively constrains erythropoiesis and granulopoiesis while preserving monocyte and macrophage compartments, thereby reshaping immune cell composition under inflammatory stress (11, 27). These lineage-specific effects indicate that iron availability acts as a contextual signal that sets developmental and activation thresholds rather than as a simple metabolic limitation. Crucially, systemic iron cues can be locally overridden through cell-intrinsic programs, allowing immune cells to decouple their iron handling from circulating iron levels when required for antimicrobial defense (12).

This signal diversification becomes particularly evident at the tissue level, where defined niches impose distinct interpretations of identical systemic cues. The spleen exemplifies how macrophage heterogeneity enables the parallel execution of iron recycling and immune surveillance: red pulp macrophages preserve iron-export functions during inflammation, whereas marginal zone macrophages prioritize pathogen capture and antigen relay but are highly sensitive to inflammatory depletion (31, 38). Such spatial partitioning allows iron recycling and immune defense to be uncoupled during infection and underscores that iron availability must be considered within tissue architectures that actively shape immune decision-making.

At the subcellular level, lysosomes emerge as an integrative platform where iron trafficking intersects with antimicrobial defense, inflammatory feedback control, and metabolic decision-making. Lysosomal iron flux can gate phagolysosomal pathogen restriction, calibrate NF-κB/IL-1β signaling, and shape mTOR-linked autophagy and metabolic programs, positioning lysosomal iron handling as a proximal signal transducer rather than a passive storage compartment.

At the cellular level, iron-dependent regulation converges on organelle function, with mitochondria emerging as a central integration point. Rather than acting as a direct inflammatory trigger, mitochondrial iron availability sets the metabolic and redox capacity within which immune signaling can occur. By constraining iron-sulfur cluster and heme synthesis, mitochondrial iron levels limit respiratory adaptation and mtROS generation, thereby gating downstream pathways such as inflammasome activation (99, 100). This framework helps explain seemingly opposing observations: iron deficiency dampens mitochondrial function, whereas iron excess amplifies inflammation by promoting redox imbalance (96, 101). These observations highlight an important unresolved question in the field: how different inflammatory microenvironments translate iron availability into pro- or anti-inflammatory signaling outcomes. Understanding how iron-sensitive pathways interact with established immune signaling cascades such as NF-κB, HIF-1α, and mTOR will be essential to determine how iron availability shapes immune responses and metabolic traits across diseases and pathologies.

Importantly, the immunometabolic consequences of iron redistribution differ across classes of pathogens. Bacterial pathogens are exquisitely sensitive to iron speciation and compartmentalization, engaging siderophore systems and iron/heme uptake pathways that are directly shaped by host iron routing decisions (126). Beyond adapting to host iron states, pathogens can actively rewire host iron signaling. For example, *Leishmania major* induces intracellular hepcidin expression and promotes Nramp1 degradation through GP63-dependent mechanisms involving altered miRNA processing, illustrating pathogen-driven manipulation of host iron-sensing pathways rather than competition for iron alone (127). Viral infections, by contrast, frequently intersect with systemic inflammation, hyperferritinemia, and dysregulated iron sequestration. Clinical and experimental studies during the COVID-19 pandemic revealed that elevated ferritin and hepcidin correlate with immune dysregulation and adverse outcomes (128–131). These findings support the concept that iron metabolism is embedded within inflammatory circuitry, rather than functioning solely as a nutritional axis or an unrelated tradeoff of a hyperinflammatory immune response. Parasitic infections, particularly malaria, further stress this framework. Hepcidin induction during *Plasmodium* infection alters iron availability across hepatic, splenic, and erythroid compartments, influencing parasite fitness, superinfection dynamics, and disease severity (132). These observations suggest that host iron redistribution shapes pathogen competition and lifecycle progression, reinforcing the need to consider iron as part of a broader host-pathogen ecological landscape.

This perspective has direct translational implications. Iron supplementation is often treated as a corrective intervention aimed at restoring erythropoiesis, yet mounting evidence indicates that iron repletion constitutes a potent immunometabolic perturbation. In infection models, iron deficiency can impair immune competence (19, 25), whereas iron supplementation in anemic hosts may exacerbate disease by increasing pathogen access to iron and amplifying inflammatory dysregulation (133, 134). Large-scale clinical analyses and intervention studies further demonstrate that the risks and benefits of iron supplementation depend on baseline iron status, inflammatory burden, and pathogen exposure, particularly in malaria-endemic regions (135, 136). These findings argue against viewing iron as a neutral nutrient and instead support a precision framework in which timing, route of administration, and inflammatory context determine immunological outcomes.

These concepts are increasingly being translated into antimicrobial strategies that exploit microbial iron dependence as a therapeutic vulnerability. Targeting or hijacking bacterial iron acquisition pathways mirrors host-driven iron sequestration and reinforces iron metabolism as a regulatory axis at the host-pathogen interface (109).

Despite substantial progress, key gaps remain. Most studies rely on systemic markers such as ferritin or transferrin saturation, whereas the immunological consequences of iron redistribution are ultimately determined by iron speciation, compartment chemistry, and organelle routing. Moreover, causal links connecting systemic iron signals to niche-specific handling, organelle function, and immune fate decisions remain incompletely defined *in vivo*. Addressing these gaps will require spatially resolved and multi-omic approaches combined with targeted perturbation of iron regulatory pathways. Such efforts will be essential to translate the emerging concept of iron as an immunometabolic signal into therapeutic strategies that modulate iron routing in inflammatory and infectious diseases.
